# Radial and Circumferential CMR-Based RV Strain Predicts Low R Wave Amplitude after ICD Implantation in Patients with Arrhythmogenic Cardiomyopathy

**DOI:** 10.3390/jcm12030886

**Published:** 2023-01-22

**Authors:** Zhongli Chen, Yanyan Song, Liang Chen, Xuan Ma, Yan Dai, Shihua Zhao, Keping Chen, Shu Zhang

**Affiliations:** 1 State Key Laboratory of Cardiovascular Disease, Cardiac Arrhythmia Center, Fuwai Hospital, National Center for Cardiovascular Disease, Chinese Academy of Medical Sciences, Peking Union Medical College, Beijing 100037, China; 2 Department of Magnetic Resonance Imaging, Fuwai Hospital, National Center for Cardiovascular Diseases, Chinese Academy of Medical Sciences, Peking Union Medical College, Beijing 100006, China; 3 State Key Laboratory of Cardiovascular Disease, Fuwai Hospital, National Center for Cardiovascular Diseases, Chinese Academy of Medical Sciences, Peking Union Medical College, Beijing 100006, China

**Keywords:** arrhythmogenic cardiomyopathy, implantable cardiac defibrillator, R wave amplitude, CMR, strain

## Abstract

Inadequate R wave amplitude (RWA) after implantable cardiac defibrillator (ICD) implantation in patients with arrhythmogenic cardiomyopathy (ACM) was suspected to relate to right ventricle impairment. However, little data-based evidence was provided to quantify the association. We retrospectively enrolled ACM patients receiving CMR examinations before transvenous ICD implantation from Fuwai Hospital. The RWA was obtained within 24 h and at 2–6-month follow-up after the operation. Structural, functional, as well as tissue characterization of the left ventricle (LV) and right ventricle (RV), were analyzed in relation to RWA. Among the 87 ACM patients (median RWA: 8.0 mV), 19 (21.8%) patients were found with low initial RWA (<5 mV) despite attempts in multiple positions. RV end diastolic diameter (RVEDD), (r = −0.44), RV ejection fraction (RVEF, r = 0.43), RV end diastolic volume index (RVEDVi, r = −0.49), RV end systolic volume index (RVESVi, r = −0.53), RV global circumferential (RVGCS, r = −0.64), and radial strain (RVGRS, r = 0.61, all *p* < 0.001) rather than LV metrics correlated strongly with initial RWA. RVGCS, RVESVi, and RVGRS were decent predictors of low RWA (areas under the curve AUC: 0.814, 0.769, 0.757, respectively) early after implantation and during 2–6-month follow-up. To summarize, low RWA of ICD lead in ACM patients was associated with RV abnormalities. The RVGCS, RVGRS, and RVESVi can be valuable predictors for identifying low RWA prior to ICD implantation.

## 1. Introduction

Arrhythmogenic cardiomyopathy (ACM) is a type of inherited adverse cardiomyopathy featuring fibrofatty replacement of the normal myocardium and holds a high risk of life-threatening ventricular arrhythmia or sudden cardiac death [1]. An implantable cardiac defibrillator (ICD) is an effective treatment with a concrete clinical base recommended by several guidelines and consensus for both the primary and secondary prevention of arrhythmic death in the high-risk population [2,3,4]. Although the ICD has demonstrated efficacy for protecting against ventricular tachycardia arrhythmias (VTA) and brings an improvement in long-term prognosis, the unfavorable sensing parameter of the ICD ventricular lead is still a major issue in the previous reports [5]. The sensing function represented by R-wave amplitude (RWA) is the cornerstone for explicit VTA identification and appropriate therapy, while a lower RWA can be either the cause of under-sensing of VTA and delayed therapy or the origination of oversensing other signals and inducer of inappropriate therapy [6,7].

Previous small-sample size studies have found that, compared with other types of progressive cardiac disease such as dilated cardiomyopathy and hypertrophic cardiomyopathy, a higher rate of low RWA (<5 mV) was observed in ARVC patients at the time of implantation and during long-term follow-up [5,8]. Another study also reported a low RWA of 7.4 mV and a significant decrease in RWA during 91 months of follow-up [9]. More significantly, in the latest study based on a multi-center registry of ACM patients, low sense values were demonstrated as the leading causes of lead-related complication, revealing that the low RWA is a major issue to be focused on and require further exploration [10]. Although previous studies all suspected that the low RWA might be associated with the extensive RV dysfunction of the disease, there is no clinical data-based evidence to quantify the relationship between the disease features and low RWA. Cardiac magnetic resonance (CMR) is an important imaging approach with accuracy and reproducibility for evaluating cardiac structure and function and has the ability for tissue characterization of fibrofatty tissue. Recent emerging CMR feature-tracking techniques also enable stable measurement of ventricular mechanics. Therefore, in this study, we aimed to investigate the clinical and cardiac imaging characteristics among the ACM patients with low RWA at implantation, analyze the determinates that affect the RWA and explore the valuable imaging markers for predicting low RWA before the ICD impanation.

## 2. Methods

### 2.1. Study Population and Definitions

We retrospectively reviewed ACM adult patients (age ≥ 18 years old at implantation) who were hospitalized in the Arrhythmia Center of Fuwai Hospital from January 2011 to March 2022 for primary implantation of ICD to prevent VTA or SCD and underwent the CMR examination within 30 days before the implantation. Among the 93 ACM patients who met the definite 2010 ARVC task force criteria (TFC) with 2 major, or 1 major plus 2 minor, or 4 minor criteria from different categories, 2 patients were excluded due to confirmed obstructive coronary heart disease and another 4 patients were excluded due to inadequate quality of CMR image. Finally, 87 consecutive ACM patients were included for analysis. Clinical history, symptoms, demographic, 12-lead or long-time monitoring electrocardiogram features, CMR, and medical treatment data were collected. Low RWA was defined as the value of the baseline rhythm R-wave voltage below 5 mV [11]. Non-sustained ventricular tachycardia (NS-VT) was defined as VT lasting for over 3 complexes but less than 30 s, while sustained VT was defined if the VT continues over 30 s. Left ventricle (LV) involvement was identified according to the previous description as one or more of the following criteria was met: LV ejection fraction (LVEF) < 50%, LV fat infiltration, or non-ischemic LV scar [12]. The study was approved by the Ethics Committee of Fuwai Hospital (IRB 2018-BG-017, Approval No. 2021-1584) and conforms to the Helsinki Declaration. Informed consent was obtained from all participants.

### 2.2. Device Implantation and Interrogation

Implanting records were reviewed to determine the device and lead type as well as the site of the RV lead position. All patients received ICD (Biotronik, Medtronic Inc. Boston Scientific, and Abbott) via a transvenous lead system by three senior electrophysiologic physicians (K.C, Y.D, and S.Z), all of the ventricular leads were active fixation leads, and were inserted in the RV apex or septum. The lead parameters, including sensing, pacing threshold, pacing impedance, and defibrillation parameters were determined during the procedure and within 24 h after the procedure. Careful attention was paid to achieve adequate sensing of 5 mV and a pacing threshold of <1.5 V per 0.5 ms, otherwise multiple endocardial positions were tested. If there was still unfavorable RWA even after several attempts, the lead was placed at a position with relatively higher sensing parameters with a shared decision by the physician and the patients. The minimum nominal sensitivity was set at 0.3 mV. The ICD parameters were repeatedly measured in 2–6-month clinic follow-up after discharge. The bipolar recordings from the right ventricular lead under intrinsic rhythm were tested repeatedly at least twice and the mean value of a stable RWA record was used for RWA comparison.

### 2.3. CMR Protocol

CMR studies were conducted on a 1.5 T MRI scanner (Magnetom Avanto, Siemens, Berlin, Germany) or a 3.0 T MRI scanner (Ingenia, Philips, Amsterdam, The Netherlands). MRI studies were transferred to offline blinded analysis. Breath-hold cine images were acquired in 3 long-axis views (LV 2-chamber, 4-chamber, and LV outflow tract) and a series of short-axis planes covering the whole LV and RV from the annulus of the aortic valves to the apex using balanced steady-state free precession sequence (b-SSFP). Cardiac volumetric and functional parameters, including left/right ventricular end-diastolic volume, left/right ventricular end-systolic volume, and left/right ventricular ejection fraction (LVEF/RVEF), were automatically generated. All the volumetric parameters were indexed to body surface area. To visually assess the fibrofatty infiltration, mDixon’s technique was applied for fat–water separation imaging [13]. Specifically, the mDixon sequence is an unbalanced rapid gradient echo sequence that achieves fat suppression through chemical shift-based water–fat separation. In addition to the in-phase/opposed-phase images of fat and water, water-only, and fat-only images can be created. The RV fat infiltration can be visualized as intramyocardial fat signals or irregular subepicardial surface of fat bordering RV [14,15] (Figure S1). The images were reviewed separately by two radiologists (Y.S and X.M) and adjudicated by the senior radiologist (S.Z) to minimize the difference.

**Figure 1:**
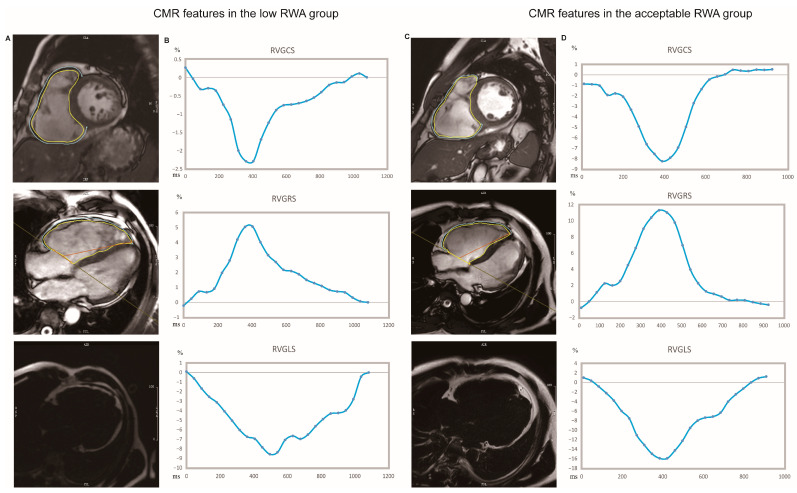
Representative CMR images of RV stain and fat infiltration from ACM patients with and without unfavorable R wave amplitude at ICD implantation. (**A**,**B**) Representative images and strain pattern from an ACM patient with low RWA (R wave amplitude of 3.6 mV at intrinsic sinus rhythm). (**C**,**D**) Representative images and strain pattern from an ACM patient with acceptable RWA (R wave amplitude of 11.7 mV at intrinsic sinus rhythm). Measurement of RV strain by CMR feature–tracking were displayed in (**A**,**C**) (upper and mid panel). The endo (yellow) and epicardial (blue) contours were delineated at the end-diastole phase. The RV global radial and circumferential strain (GCS, GRS) were obtained from the short-axis ((**A**,**C**) Upper panel), while the global longitudinal strain was measured from 4-chamber view ((**A**,**C**) mid), respectively. The lower peak circumferential ((**B**,**D**) upper panel), radial ((**B**,**D**) mid panel), and longitudinal ((**B**,**D**) lower panel) strain of the right ventricle demonstrated more severe abnormalities of the RV strain in the patient with final low RWA. CMR images from mDixon’s water-fat separation imaging were displayed at (**A**,**C**) (lower panel).

Late gadolinium enhancement (LGE) images were acquired 10 to 15 min after intravenous administration of gadolinium-DTPA (Magnevist, Bayer, Berlin, Germany) at a dose of 0.2 mmol/kg with a breath-held phase-sensitive segmented inversion-recovery sequence in the same views as the cine images. The presence of LGE in LV myocardium was visually analyzed using the American Heart Association (AHA) 16-segment model by consensus reading of 2 independent observers (Y.S and X.M). The regions of LGE were fine-tuned by the operator to reduce false-positive when necessary. CMR-FT analysis was carried out by using CVi42 (Circle Cardiovascular Imaging Inc, Calgary, AB, Canada). LV and RV end-diastolic endocardial and epicardial contours were traced semi-automatically in long-axis views (two-chamber, three-chamber, and four-chamber) and short-axis view on cine images by investigators blinded to the clinical and CMR data. All contours were adjusted after visual inspection during cine loop playback to ensure appropriate tracking of LV and RV segments. The global longitudinal strain (GLS) was obtained from the 4-chamber view for the RV and from the 2-chamber, 3-chamber, and 4-chamber views for the LV. The global circumferential and radial strain (GCS and GRS) parameters for both the RV and the LV were determined in the short-axis views of cine images [16]. And the delineated RV endocardial and epicardial borders for GLS, GCS, and GRS measurement were presented in Figure 1A, C.

### 2.4. Statistical Analysis

Continuous variables distributions were tested for normality via the Shapiro–Wilk test and were described as mean with standard deviation if normally distributed, otherwise as median with interquartile range (IQR). The discrete variables were depicted as counts and proportions. Student’s *t*-test/Mann–Whitney U test, analysis of variance, or Kruskal–Wallis test was applied for evaluating the differences between continuous variables as appropriate, and Dunn’s multiple comparisons were used for post hoc analysis. The chi-square test or Fisher’s exact test was used for assessing the difference of discrete variables between groups. The simple correlation between continuous imaging markers and RWA was examined by the Spearman correlation rank test and evaluated by the correlation coefficient and *p*-value. Univariate linear regression analysis was also performed to assess the clinical and imaging variables associated with RWA with parameter estimates and standard errors reported. The independent associations between RV mechanical, tissue characterization variables, and RWA were further accessed by adjusting for demographic, ECG, and traditional structural and function imaging features and lead position using a multivariate linear regression model. The independence of variables was confirmed by the Durbin–Watson statistic. A value of Durbin–Watson statistic close to 2 indicates no autocorrelation within the model [17]. The receiver operating characteristic (ROC) curves were performed to evaluate the discriminability of each imaging marker for predicting low RWA in all ACM patients and the LV involvement subgroup. The optimal cutoff value for each imaging marker was calculated based on the highest Youden’s Index (sensitivity + specificity − 1) in all patients and was applied in the LV involvement subgroup for sensitivity and specificity assessment. The reproducibility of the CMR-derived RV strain and tissue characteristics were evaluated by the Bland–Altman plot and intraclass correlation coefficient (ICC). All tests were 2-tailed with an α level of 0.05 considered statistically significant. Statistical analyses were performed using R software version 4.1.2 and SPSS version 22 (IBM, Armonk, NY, USA).

## 3. Results

### 3.1. Baseline Characteristics

A total of 87 patients were included for final analysis, of whom 77% were implanted for secondary prevention while the rest 23% were implanted for primary prevention. The mean age at initial ICD implantation was 43.8 years old and 70.1% were male patients. For these patients, genetic testing was performed on 15, and 10 of them were positive for pathogenic variants, with PKP2 (*n* = 5) and DSG2 (*n* = 5) as the most common pathogenic gene. The median RWA was 8.00 mV (IQR: 5.1–11.8 mV) in the total population. In 19 of the 87 patients, though multiple positions of the RV lead were tested for achieving adequate pacing parameters, the final RWA achieved in ARVC remained lower than 5 mV.

The baseline clinical and pacing characteristics of the total population as well as a comparison between those with and without low RWA were described in Table 1. Demographic features and clinical symptoms did not differ in those with acceptable RWA and low RWA. Compared with patients with acceptable RWA, those with low RWA did not display more severe HF symptoms but held prominent cardiac arrhythmias. NYHA status in those with low RWA was not that severe, but rather better, with 73.7% in NYHA class I-II while a majority with acceptable RWA was mainly distributed in NYHA class III-IV. Patients with low RWA displayed a higher proportion of T-wave inversion in V1-V3 (94.7%) lead, while bundle branch block and epsilon waves showed no significant difference between the two groups. Compared with those with acceptable RWA, those with low RWA had higher proportion history of NS-VT (94.7% vs. 55.8%), RV-originated or left bundle branch block (LBBB) morphology VT (100% vs. 47.1%), and a relatively heavier burden of 24 h ambulatory monitoring PVC counts (median: 3056 vs. 745). Compared with those having acceptable RWA, a lower proportion of patients in the RWA group received diuretic agents and renin–angiotensin–aldosterone system inhibitors while usage of anti-arrhythmia drugs was more frequent in this group, though the difference was not statistically significant.

In terms of ICD-related parameters, the median RWA in the low RWA and acceptable RWA group was 3.1 mV (IQR (2.7–4.2 mV)) and 9.7 mV (IQR (7.3–13.6) mV), respectively, while the pacing threshold and impedance were satisfactory and did not differ between the two groups. Furthermore, there is no difference in the ICD brands and ICD types between those presented with low RWA or acceptable RWA. The final screwed lead position of the low RWA group was more frequent in RV apex compared with the acceptable RWA group, but this does not reach statistical significance.

### 3.2. CMR-Based Structural and Functional Characteristics

Biventricular morphology and function parameters were calculated, and the regional motion was evaluated using CMR-FT. Tissue characterization was depicted by LGE and fat infiltration. Table 2 details these structural and functional features in the total population and the differences in CMR parameters between the low RWA and acceptable RWA groups. Patients with lower RWA had more extensively impaired RV structure and function, with more pronounced RV enlargement and particularly lower right ventricular ejection fraction (RVEF). Synergistically, RV radial (5.10 (3.31–6.98) vs. 9.87 (5.40–14.76), *p* = 0.002) and circumferential strain values (−2.86 (−4.17–1.95) vs. −6.08 (−9.42–−3.52), *p* < 0.001) were significantly impaired in the low RWA group, as compared to the other group (Figure 1). The presence of RVLGE and fat infiltration was higher in the low RWA versus the acceptable RWA group, indicating a more prominent myocardium injury. However, there was no significant difference in LV structural or functional metrics between the two groups.

### 3.3. The Clinical, Operation, and Imaging Correlates of RWA among ACM Patients

**Figure 2:**
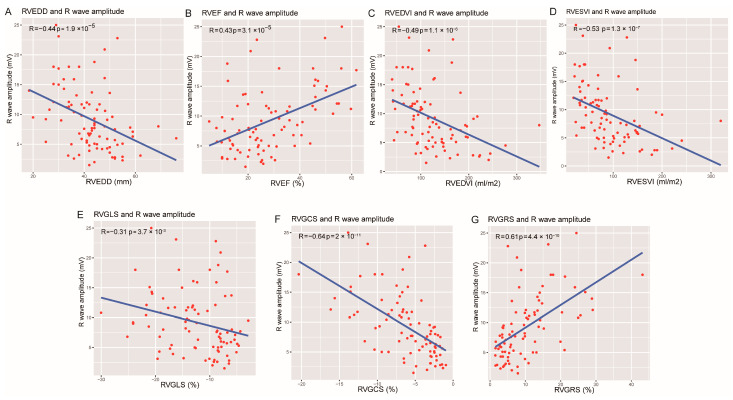
The linear relationship between right ventricular structural, functional strain parameters, and R wave amplitude. (**A**–**D**) Correlation between right ventricular (RV) ejection fraction, RV end-diastolic diameter, RV end-diastolic volume index, RV end-systolic volume index, and R wave amplitude (RWA) of ICD lead. (**E**–**G**) Correlation between RV global longitudinal, circumferential and radial strain, and RWA.

A lone RV presentation was found in 44 patients, biventricular involvement in 30 patients, and LV-dominant in 13 patients. RWA was significantly lower among the RV lone and biventricular involvement ACM group while no low RWA was found present in the LV-dominated group (Figure S2A). Compared with those with ICD leads implanted in the RV apex, RWA was higher in those with septal RV lead placement, while no difference or correlation was observed between RWA and different ICD brands, pacing threshold, or impendence (Figure S2B–E). Univariate linear regression revealed that, among the clinical and traditional parameters, ICD RWA was associated with the presence of TWI in V1-V3 lead, Epsilon wave, LBBB morphology or RV-originated VT, RVEF, right ventricular end-diastolic diameter (RVEDD), right ventricular end-systolic volume index (RVESVi) and right ventricular end-diastolic volume index (RVEDVi), but displayed mild association with left ventricular end-diastolic diameter (LVEDD), left ventricular end-diastolic volume index (LVEDVi) and left ventricular end-systolic volume index (LVESVi) and non-significant association with age, sex, body mass index, right bundle branch block pattern or LVEF (Table 3). Ventricular structural, functional, and motion parameters provided by CMR, the RV metrics including RVEDD (r = −0.44, *p* < 0.001), RVEF (r = 0.43, *p* < 0.001), RVESVi (r = −0.53, *p* < 0.001), RVEDVi (r = 0.49, *p* < 0.001), RVGLS (r = −0.31, *p* < 0.001), RVGRS (r = 0.61, *p* < 0.001), and RVGCS (r = 0.64, *p* < 0.001), rather than the LV features (Figure S3), were strongly correlated with RWA (Figure 2). And multivariate linear regression reveals that RVGRS and RVGCS were still strong determinates of RWA independent of other clinical and traditional imaging parameters (Table 3).

### 3.4. CMR Imaging Predictors for Identifying Low RWA Risk

**Figure 3:**
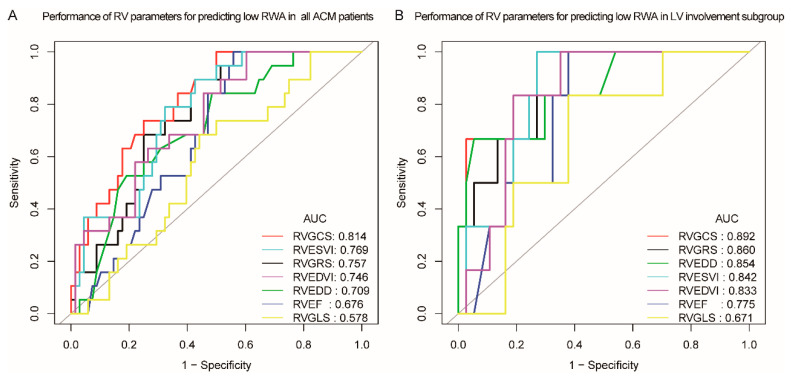
Receiver operating curves of RV parameters and low RWA. (**A**) Receiver operating curves showing the predictive performance of RV parameters in identifying patients with low RWA after ICD implantation in all ACM patients. (**B**) Receiver operating curves showing the predictive performance of RV parameters in identifying patients with low RWA after ICD implantation in ACM patients with LV involvement.

The performance of CMR imaging markers for predicting low RWA after ICD implantation was evaluated by ROC analysis. The AUC with 95% CI, specificity, and sensitivity with the best cut-off values were listed in Table 4. In the functional and morphological parameters, RVEDD and RVEF only held moderate predictive ability while RVESVi, RVGCS, and RVGRS demonstrated more decent predictive values with AUC of 0. 814, 0.769, and 0.757 respectively. However, the RVGLS demonstrates poor ability with lower CI limits of less than 0.5 (Table 4 and Figure 3A). Similar observations were found in the LV-involvement subgroup, with the RVGCS, RVESVi, and RVGRS still holding good performance for predicting low RWA (AUC: 0.892, 0.842, 0.860) (Table S1 and Figure 3B).

### 3.5. Association of Pre-Procedure CMR Parameters and RWA at 2–6-Month Follow-Up

At first follow-up after patients discharge, generally at 2–6 months post-implantation. The median initial RWA measured within 24 h after ICD implantation through the device was not different compared with that measured at 2–6 months (8.00 (5.10–11.85) mV vs. 7.80 (4.80–11.80) mV, *p* = 0.165). All patients with RWA lower than 5 mV remained having low RWA level. Four (5.9%) patients in acceptable RWA group at implantation have RWA dropped to lower than 5 mV at the first follow-up. There is a slight increase in pacing threshold (0.50 (0.50–0.80) V @0.5 ms vs. 0.75 (0.50–1.00) V @0.5 ms, *p* < 0.001). The median pacing impedance measured at implantation decreased from 595.0 (IQR 509.5–774.0) to 550.0 (IQR: 464.0–668.0 ohms). The RV parameters derived from CMR analysis was also strongly correlated with the follow-up RWA, while the LV metrics did not display significant correlation (Table S3). The RVGCS, RVGRS, and RVESVi still held strongest association with low RWA at 2–6-month follow-up (RVGCS, r = −0.66; RVGRS, r = 0.63; RVESVi, r = −0.58, all *p* < 0.001), these parameters still held high predictive values for low RWA at 2–6-month follow-up (AUC: 0.824, 0.783, 0.812).

### 3.6. Intra- and Interobserver Variability

**Figure 4:**
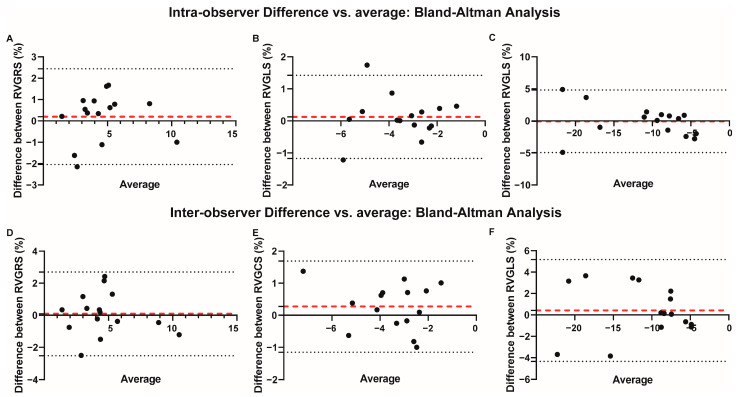
Bland–Altman analysis of RV strain for intra- and inter-observer variability. (**A**–**C**) Variability of intra-observer for RVGRS, RVGLS and RVGCS quantification. (**D**–**F**) Variability of inter-observer for RVGRS, RVGLS and RVGCS quantification. Red dashed line: bias error; black dashed lines: 95% limits of agreement.

The inter-observer and intra-observer variability of RV strain parameters were displayed in Table S2 and Figure 4. The intra-observer ICC was 0.886 (95% CI: 0.699–0.960) for RVGRS, 0.889 (95% CI: 0.731–0.965) for RVGCS, and 0.932 (95% CI: 0.785–0.973) for RVGLS, indicating satisfactory reproducibility. Inter-observer measurement also showed good agreement, with ICC over 0.8 for these parameters.

## 4. Discussion

In this study, we observed pronounced arrhythmia clinical phenotypes rather than more severe LV dysfunctions in those with low RWA and we also found it is the impaired RV structure and function rather than the LV measurement correlate with the RWA. Specifically, abnormal morphological parameters such as RVEF, RVEDD, RVEDVi, RVESVi, impaired RV mechanics, including RVGLS, RVGCS, and RVGRS, as well as the presence of RV scar and fat infiltration were all associated with lower RWA within 24 h after ICD implantation. The RVESVi, RVGCS, and RVGRS held a good performance for differentiating low RWA in all ACM patients and the LV involvement subgroup and they also held predictive value of low RWA at 2–6-month follow-up after ICD implantation. Therefore, the RVGCS, RVGRS, and RVESVi derived from CMR are valuable parameters for predicting low RWA before the ICD implantation and might assist in pre-procedural planning, the decision of additional intraoperative mapping or optimized position for lead placement in ACM patients.

### 4.1. Issue of Low RWA after ICD Implantation in ACM

Although recent years have witnessed the benefit brought by ICD for reducing the rate of SCD and improving the prognosis in ACM patients [18], low sensing parameters during ICD implantation were still an unignorable issue as it is closely related to ICD complications such as under-sensing of VT and T-wave oversensing [7,10]. In this study, despite the usage of screw-in active leads, and multiple attempts of RV sites, a lower final RWA still existed in 19 of 87 patients, and the mean RWA was relatively low at 8.0 mV. Additionally, in the 19 patients with initial low RWA we found they still have RWA at a low level at 2–6-month follow-up. In fact, among these patients, we observed three device-related complications from our center after a median follow-up of 36 months. One patient underwent inappropriate ICD shocks due to oversensing while the other two patients with very low RWA (2.6 mV and 3.1 mV) experienced new ventricular lead implantation because of huge increase in impedance and loss of capture after one year and three years, respectively. A similar observation was also reported by Link, Wichter, and Mugnai in earlier studies [9,19,20], thus re-emphasizing this issue in ICD implantation among ARVC patients. But the factors that contributed to the low RWA among the ACM patients have not been analyzed or proved by clinical or imaging data. As a progressive disease predominantly affecting the right ventricle, earlier in 1994, it has been suspected that the diseased RV myocardium might affect the sensing parameter and increase the difficulty of lead placement, especially in patients with extensive right structure abnormality [20]. But to date, the valuable parameter to quantify the RV disease severity and help to identify the probability of unfavorable RWA is still lacking. Our study is the first to quantitively evaluate the ACM disease features and their relationship with RWA based on clinical characteristics and CMR imaging data. In this study, we included ACM patients with significant RV dysfunction (mean RVEF of 28.24 ± 14.19%) in whom the majority were implanted for secondary prevention of SCD. Though the low RWA group held slighter HF symptoms, and seemingly better LV structure and function, the cardiac arrhythmia features were quite pronounced in the low RWA group compared with the high RWA group, which might contribute to the vicious circle that renders the appropriate and timely VA therapy in the group who might potentially benefit more from ICD, thus reinforcing low RWA as an important problem relating to ICD implantation in ACM patients.

### 4.2. Relationship between RWA and ACM

Regarding the device or operation-related factors, we did not observe a significant difference in the device type, device brand, or pacing threshold and impedance between the acceptable RWA and low RWA groups. And among our population, the pacing thresholds were all below 1.5 V at 0.5 ms and impedance within the normal range, without dislocation events reported within 24 h after ICD implantation or before patients discharge.

Furthermore, regarding the RWA the first follow-up after discharge during 2–6 months, the overall RWA of remained stable (8.00 (5.10–11.85) mV vs. 7.80 (4.80–11.80) mV, *p* = 0.165) at 2–6 months compared with the value measured 24 h after implantation, but the pacing threshold was slightly increased, with impedance decreased (Figure S4A–C), which is similar to the previous findings [21]. Those with low RWA at first interrogation after implantation still have low RWA. Therefore, it is reasonable to suspect that the low sensing parameter was more likely to be associated with the disease nature due to diseased RV myocardium rather than the acute injury from procedure of ICD device implantation.

CMR allows for accurate quantification of biventricular morphology and function and provides additional information on tissue characterization. In the CMR-derived structural and functional parameters, larger RVEDD, lower RVEF, higher RVESVi, and RVEDVi, rather than corresponding LV parameters, were all strongly correlated with lower RWA. Additionally, the unfavorable RWA was absent from LV-dominated ACM phenotype and those with RVEF over 40%, which further affirms the dominating effect of abnormality from RV on the low sensing values of ICD.

In addition to the traditional morphological parameters, the myocardium impairment can be more directly analyzed by both the fibrosis/fibrofatty replacement and regional motion abnormality. These factors are also emphasized in the diagnostic TFC as a visual assessment of RV regional wall motion abnormalities and histopathological findings. However, the visual assessment of RV motion is subjective and myocardial biopsy can be limited by the sample location and number. Even though direct assessment of fibrofatty replacement was challenging due to the thin RV structure, some advances in CMR imaging have been achieved. The emerging feature tracking technique based on SSFP images, the mDixon technique of fat–water separation, and the classical LGE analysis may provide a new modality for objective quantification of wall motion or noninvasive visual assessment of tissue characterization [22,23]. Previous studies have indicated the reproducibility of the strain analysis by CMR-FT as well as the application of mDixon in fat infiltration identification and revealed that RV strain and fat infiltration might be clinically important due to the association with ventricular arrhythmias [22,24]. And herein we found that the RV strain, LGE, and fat infiltration might also be correlates of the ICD sensing parameter. By contrast, the association between LV mechanics and LGE and RWA is lacking. This was also reaffirmed by the absence of low RWA in LV-dominated phenotype, in which there is no severe RV infiltration. Therefore, it may be concluded that in ACM patients who have an indication for ICD implantation, those with involvement of RV abnormities rather than the LV-dominated phenotype warrants more pre-procedure planning regarding the risk of unfavorable RWA. Furthermore, we also found the RVGCS and RVGRS were associated with RWA independent of both the clinical features and the morphological parameters (RVGCS beta: −0.608; RVGRS beta: 0.291). These findings provided data-based clinical evidence for RV impairment as the determinant of the RWA of ICD and also indicate that apart from the morphology, the novel metrics from the CMR were critical contributors and predictors of the unsatisfactory sensing parameters.

RVEDD and RVEF were classical routine parameters for evaluating RV abnormality, but for predicting the low RWA probability, the discriminability of the two parameters is not as good as RVESVi and RVEDVi, which held AUC of 0.769 and 0.746, respectively. More importantly, CMR-FT-derived novel strain parameters such as RVGCS and RVGRS provided decent performance for predicting low RWA within 24 h after implantation with higher AUC in both the overall ACM population (0.814, 0.757) and those with LV involvement (0.892, 0.860), and these metrics are also capable of predicting low RWA at first follow-up at 2–6 months, which means that by resorting to the CMR evaluation before the ICD implantation, one might predict the possibility of low RWA in the ACM patients.

## 5. Clinical Implications

Despite the fact that ICD is an undouble effective treatment for VA and SCD prevention in ACM patients, the low RWA is a troubling issue that adds difficulties to lead placement and leaves a risk for future complications such as delay or inappropriate therapy. Therefore, knowing which kind of ACM patient was more likely to experience low RWA at ICD implantation allows for a more thorough preparation of the operation. The current study offers a concept in the explanation of potential underlying factors responsible for low RWA after ICD implantation in ACM utilizing CMR to assess morphological and functional parameters and provides the possibility to predict tough situations preoperatively, thus advancing operational planning. Encouragingly, during recent years, more promising attempts have been made for dealing with the problem such as clinical usage of subcutaneous ICD [25], electrical voltage mapping for navigating lead placement, placing the sensing lead in LV epicardium or LV septal in patients with low RWA for RV lead placement [26,27,28]. By referring to the simple RV parameters (RVGCS, RVGRS, and RVESVi) from the non-contrast cine imaging of CMR, operators might find it easier to identify the ACM patients who are at high risk of low final RWA and who might be possible candidates for this new approach for rescue. The operators and patients might have more space to take the initiative for thorough shared decision-making of the ICD choice and selection of intraoperative procedure or navigation techniques. However, to date, the CMR RV strain analysis and the rescue procedure approaches are still under research but not included in clinical routine; there is still a long way to go for appropriately solving the problem of low RWA of ICD among ACM patients. Additionally, more clinical and imaging studies are warranted for further validation of the new parameters and techniques.

## 6. Limitations

This study also has several limitations. Firstly, as a retrospective study based on single-center experience, the detailed records of intraoperative parameters during every attempted position were not available, and information on difficulties in lead placement was described quantitively in the implanter notes so we cannot calculate the explicit attempted positions for each patient. Secondly, despite RV strain as a promising parameter for quantifying RV dysfunction, there are still no standardized normal values for the strain values, so it is still a research tool and not routinely applied in clinical practice. Additionally, although the CMR-FT is a less time-consuming and user-friendly method for RV analysis [29], compared with the CMR tagging technique, the results can also be limited by the single post-processing FT technique, which lacks interstudy agreement compared with the fast strain-encoded cardiovascular magnetic resonance imaging [23]. Future studies should further evaluate the reproductivity, sensitivity, and specificity of the results through different strain analysis techniques. Additionally, due to the challenge of interpreting the presence of RV fat infiltration in the thin RV structure, this metric is subjective and less reliable than other structural and functional parameters. Finally, though the study population is relatively higher than in previous ones, the small sample size and a small number of low RWA events still limit the statistical power of detecting differences and the opportunities for multivariable modeling.

## 7. Conclusions

In conclusion, the structural, functional, mechanical, and tissue impairments of RV affect the RWA after ICD implantation. The RVESVi as well as RV strains including RVGRS and RVGCS provided by CMR were promising imaging markers for predicting low RWA and might assist in the preoperative planning before ICD implantation.

## Figures and Tables

**Table 1:** Baseline features and ICD parameters of ACM patients with and without low RWA.

	All (*n* = 87)	Acceptable RWA (*n* = 68)	Low RWA (*n* = 19)	*p*-Value
Age at implantation, yrs	43.8 ± 14.3	43.3 ± 13.3	41.0 ±17.8	0.542
Male, n (%)	61 (70.1%)	47 (69.1%)	14 (73.7%)	0.701
BMI, kg/m^2^	24.1 ± 3.6	24.2 ± 3.8	24.0 ± 3.1	0.845
NYHA Class, n (%)				0.006
I	2 (2.3%)	2 (2.9%)	0 (0.00%)	
II	34 (39.1%)	20 (29.4%)	14 (73.7%)	
III	38 (43.7%)	34 (50.0%)	4 (21.0%)	
IV	13 (14.9%)	12 (17.7%)	1 (5.3%)	
Family History of ACM, n (%)	12 (13.8%)	9 (13.24%)	3 (15.8%)	0.775
**Symptoms**				
Syncope, n (%)	44 (50.6%)	34 (50.0%)	10 (52.6%)	0.839
Amaurosis, n (%)	31 (35.6%)	22 (32.4%)	9 (47.4%)	0.227
Chest pain, n (%)	7 (8.1%)	7 (10.3%)	0 (0.00%)	0.339
Fatigue, n (%)	24 (27.6%)	20 (29.4%)	4 (21.1%)	0.471
Stuffy, n (%)	46 (52.9%)	36 (52.9%)	10 (52.6%)	0.981
Palpation, n (%)	66 (75.9%)	51 (75.0%)	15 (79.0%)	0.722
Oedema, n (%)	6 (6.9%)	5 (7.4%)	1 (5.3%)	1.000
**ECG Characteristics**				
TWI: V1-V3, n (%)	56 (64.4%)	38 (55.9%)	18 (94.7%)	0.002
TWI: V4-V6, n (%)	34 (39.1%)	26 (38.2%)	8 (42.1%)	0.760
RBBB pattern, n (%)	28 (32.2%)	20 (29.4%)	8 (42.1%)	0.295
LBBB pattern, n (%)	0 (0%)	0 (0%)	0 (0%)	-
Epsilon Wave, n (%)	23 (26.4%)	15 (22.1%)	8 (42.1%)	0.080
**Arrhythmia Characteristics**				
History of sustained SVT/VF, n (%)	67 (77.0%)	53 (77.9%)	14 (73.7%)	0.697
NS-VT, n (%)	56 (64.4%)	38 (55.8%)	18 (94.7%)	0.002
24 h PVC counts, n (n = 72)	880 (87–3550)	754 (1–3158)	3056 (405–10,000)	0.067
RV originated /LBBB morphology VT, n (%)	51 (58.6%)	32 (47.1%)	19 (100.00%)	<0.001
LV originated/RBBB morphology VT, n (%)	19 (21.8%)	16 (23.5%)	3 (15.8%)	0.470
Multi-morphology PVC, n (%) (n = 72)	49 (68.1%)	35 (63.6%)	14 (82.4%)	0.148
ICD Brand				0.631
Biotronik, n (%)	34 (39.1%)	26 (38.2%)	8 (42.1%)	
Boston Scientific, n (%)	12 (13.8%)	11 (16.2%)	1 (5.3%)	
Medtronic, n (%)	19 (21.8%)	15 (22.1%)	4 (21.1%)	
Abbott, n (%)	22 (25.3%)	16 (23.5%)	6 (31.6%)	
ICD Type				0.391
Single-chamber, n (%)	66 (75.9%)	53 (77.9%)	13 (68.4%)	
Dual-chamber, n (%)	21 (24.1%)	15 (22.1%)	6 (31.6%)	
Ventricular Lead position				0.164
Apex, n (%)	57 (65.5%)	42 (61.8%)	15 (78.9%)	
Septum, n (%)	30 (34.5%)	26 (38.2%)	4 (21.1%)	
**Pacing parameters**				
R wave amplitude, mV	8.0 (5.1–11.8)	9.7 (7.0–13.6)	3.1 (2.7–4.2)	<0.001
Impedance, ohms	595.0 (509.5–774.0)	595.5 (520.0–781.0)	560.0 (493.0–753.5)	0.332
Pacing threshold, V @0.5 ms	0.5 (0.5–0.8)	0.5 (0.5–0.8)	0.5 (0.5–0.7)	0.645
**Medication Treatment**				
Diuretic agent, n (%)	42 (48.3%)	35 (51.5%)	7 (36.8%)	0.259
ACEI/ARB/ARNI, n (%)	47 (54.0%)	40 (58.8%)	7 (36.8%)	0.089
AAD, n (%)	58 (66.7%)	43 (63.2%)	15 (78.9%)	0.199
Beta Blocker, n (%)	77 (88.5%)	59 (86.8%)	18 (94.7%)	0.335

Notes: BMI: body mass index; NYHA: New York Heart Association; ACM: arrhythmogenic cardiomyopathy; ECG: electrocardiogram; TWI,: T-wave inversion; RBBB: right bundle branch block; LBBB: left bundle branch block; SVT: sustained ventricular tachycardia; VF: ventricular fibrillation; NS-VT: non-sustained ventricular tachycardia; RV: right ventricle; LV: left ventricle; PVC: premature ventricular contraction; ACEI: angiotensin converting enzyme inhibitor; ARB: angiotensin receptor blocker; ARNI: angiotensin receptor–neprilysin inhibitor; AAD: anti-arrhythmia drug.

**Table 2:** Ventricular morphology and function evaluated by CMR.

	All (*n* = 87)	Acceptable RWA (*n* = 68)	Low RWA (*n* = 19)	*p*-Value
**Right Ventricular**				
RVEDD, mm	43.40 ± 10.25	41.03 ± 10.59	47.30 ± 7.23	0.018
RVEF, %	28.24 ± 14.19	30.35 ± 15.03	20.70 ± 6.74	0.008
RVEDVi, mL/m^2^	115.33 (83.50–161.51)	102.32 (80.42–139.41)	147.08 (112.48–215.41)	0.001
RVESVi, mL/m^2^	81.81 (46.70–125.07)	70.14 (40.52–111.97)	113.54 (92.36–168.94)	<0.001
RVGLS, %	−12.09 (−16.59-−8.00)	−12.58 (−17.19-−8.01)	−9.23 (−13.70–−7.81)	0.299
RVGCS, %	−5.56 (−8.31-−2.95)	−6.08 (−9.42–−3.52)	−2.86 (−4.17–1.95)	<0.001
RVGRS, %	7.88 (4.17–13.61)	9.87 (5.40–14.76)	5.10 (3.31–6.98)	0.002
RVLGE presence, n (%)	54 (62.07%)	36 (52.94%)	18 (94.74%)	<0.001
RV-FAT infiltration, n (%)	50 (57.47%)	32 (47.06%)	18 (94.74%)	<0.001
**Left Ventricular**				
LVEDD, mm	52.90 ± 9.40	53.38 ± 10.30	51.17 ± 3.00	0.367
LVEF, %	46.62 ± 13.24	45.65 ± 13.76	50.09 ±10.83	0.198
LVEDVi, mL/m^2^	82.70 (63.25–105.70)	83.04 (66.91–110.22)	79.87 (59.92–98.55)	0.377
LVESVi, mL/m^2^	42.31 (26.31–64.91)	42.34 (26.44–68.66)	36.61 (25.60–58.81)	0.309
LVGLS, %	−11.49 ± 3.40	−11.50 ± 3.47	−11.46 ± 3.21	0.964
LVGCS, %	−15.14 (−17.50-−10.91)	−14.87 (−17.01-−10.49)	−15.98 (−18.31-−13.82)	0.169
LVGRS, %	23.03 ± 9.56	22.33 ± 9.63	25.52 ± 9.12	0.200
Positive LV-LGE, n (%)	44 (50.57%)	34 (50.00%)	10 (52.63%)	0.839
LV-LGE extend (%)	6.72 (0.00–18.47)	3.36 (0.00–18.80)	7.22 (0.00–14.21)	0.751

Notes: RVEDD: right ventricular end diastolic diameter; RVEF: right ventricular ejection fraction; RVEDVi: right ventricular end diastolic volume index; RVESVi: right ventricular end-systolic volume index; RVGL (C/R) S: right ventricular global longitudinal, circumferential, radial strain; LVEDD: left ventricular end-diastolic diameter; LVEF: left ventricular ejection fraction; LVEDVi: left ventricular end-diastolic volume index; LVESVi: left ventricular end-systolic volume index; LVGL (C/R) S: left ventricular global longitudinal, circumferential, radial strain; LGE: late gadolinium enhancement.

**Table 3:** Determinant factors of R wave amplitude after ICD implantation.

	Univariate Analysis		Multivariate Analysis	
	Beta (SE)	*p*-Value	Beta (SE)	*p*-Value
Age, yrs	0.048 (0.040)	0.229		
Male, (vs. female)	0.229 (1.253)	0.855		
BMI, kg/m^2^	0.083 (0.159)	0.602		
Epsilon	−2.436 (1.274)	0.059		
RBBB	−1.654 (1.215)	0.177		
TWI: V1-V3	−3.530 (1.135)	0.003		
LBBB-VT	−3.992 (1.081)	<0.001		
LVEF, %	−0.048 (0.043)	0.267		
LVEDD, mm	0.130 (0.060)	0.033		
LVESVi, mL/m^2^	0.037 (0.020)	0.067		
LVEDVi, mL/m^2^	0.034 (0.018)	0.063		
RVEF, %	0.180 (0.036)	<0.001		
RVEDD, mm	−0.205 (0.052)	<0.001		
RVESVi, mL/m^2^	−0.040 (0.009)	<0.001		
RVEDVi, mL/m^2^	−0.038 (0.009)	<0.001		
**CMR-FT and tissue characterization**				
LVGLS, %	0.049 (0.170)	0.772		
LVGCS, %	0.159 (0.128)	0.217		
LVGRS, %	−0.055 (0.060)	0.362		
LV-LGE	1.002 (1.142)	0.383		
LV-LGE extent, %	0.061 (0.038)	0.109		
RVGLS, %	−0.379 (0.061)	0.015	−0.085 (0.106)	0.455 ^a^
RVGCS, %	−0.771 (0.115)	<0.001	−0.608 (0.183)	0.001 ^b^
RVGRS, %	0.356 (0.062)	<0.001	0.291 (0.097)	0.004 ^c^
RV-LGE	−4.115 (1.095)	<0.001	−0.558 (1.466)	0.705 ^d^
RV-FAT	−4.022 (1.075)	<0.001	−1.363 (1.289)	0.294 ^e^

Notes: RBBB: right bundle branch block; LBBB: left bundle branch block; VT: ventricular tachycardia; TWI:V1–V3: T-wave inversion in V1–V3 lead of 12 lead electrocardiogram; RVEDD: right ventricular end-diastolic diameter; RVEF: right ventricular ejection fraction; RVEDVi: right ventricular end-diastolic volume index; RVESVi: right ventricular end-systolic volume index; RVGL (C/R) S: right ventricular global longitudinal, circumferential, radial strain; LVEDD: left ventricular end-diastolic diameter; LVEF: left ventricular ejection fraction; LVEDVi: left ventricular end-diastolic volume index; LVESVi: left ventricular end-systolic volume index; LVGL (C/R) S: left ventricular global longitudinal, circumferential, radial strain; LGE: late gadolinium enhancement. Multivariate analysis adjusted for age, sex, TWI (V1–V3), LBBB morphology VT, LVEF, LVEDD, RVEF, RVEDD, Epsilon, lead position; ^a^: adjusted R square 0.241, Durbin–Watson test statistics 1.741; ^b^: adjusted R square 0.336, Durbin–Watson test statistics 1.779; ^c^: adjusted R square 0.320, Durbin–Watson test statistics 1.864; ^d^: adjusted R square 0.239, Durbin–Watson test statistics 1.750; ^e^: adjusted R square 0.249, Durbin–Watson test statistics 1.79.

**Table 4:** Imaging parameters for predicting Low RWA.

Variables	ROC Area (AUC)	95% CI Low	95% CI Upper	Best Cutoff	Sensitivity	Specificity
RVEDD, mm	0.709	0.585	0.832	41.8	0.842	0.515
RVEF, %	0.677	0.563	0.790	31.5	1.000	0.441
RVEDVi, ml/m^2^	0.746	0.631	0.861	88.5	1.000	0.397
RVESVi, mL/m^2^	0.769	0.664	0.875	78.4	0.895	0.574
RVGRS, %	0.757	0.653	0.861	8.75	0.895	0.574
RVGCS, %	0.814	0.718	0.910	−6.55	1.000	0.500
RVGLS, %	0.578	0.443	0.713	−11.43	0.684	0.559

Notes: RVEDD: right ventricular end diastolic diameter; RVEF: right ventricular ejection fraction; RVEDVi: right ventricular end diastolic volume index; RVESVi: right ventricular end-systolic volume index; RVGL (C/R) S: right ventricular global longitudinal, circumferential, radial strain; AUC: area under receiver operative curve; CI: confidence interval.

## Data Availability

Data supporting this study are available upon reasonable request to the corresponding author.

## Supplementary Materials

The following supporting information can be downloaded at: https://www.mdpi.com/article/10.3390/jcm12030886/s1, Figure S1: An example of mDixon images for RV fat identification; Figure S2: Relationship between R wave amplitude, ACM subtypes, lead position, ICD brands, and pacing parameters; Figure S3: Relationship between left ventricular parameters and R wave amplitude; Figure S4: Changes of pacing parameters at 2–6-month follow-up and receiver operating curves of RV parameters and low RWA at 2–6 months; Table S1: Imaging parameters for predicting Low RWA in LV involvement subgroup; Table S2: Reproducibility for CMR-derived novel right ventricular strain parameters; Table S3: Correlation between right ventricular (RV) and left ventricular (LV) metrics and R wave amplitude (RWA) at 2–6-month follow-up.

## References

[1] Corrado D., Link M.S., Calkins H. (2017). Arrhythmogenic Right Ventricular Cardiomyopathy. N. Engl. J. Med..

[2] Towbin J.A., McKenna W.J., Abrams D.J., Ackerman M.J., Calkins H., Darrieux F.C., Zareba W. (2019). 2019 HRS expert consensus statement on evaluation, risk stratification, and management of arrhythmogenic cardiomyopathy: Executive summary. Heart Rhythm..

[3] Corrado D., Wichter T., Link M.S., Hauer R.N., Marchlinski F.E., Anastasakis A., Calkins H. (2015). Treatment of Arrhythmogenic Right Ventricular Cardiomyopathy/Dysplasia: An International Task Force Consensus Statement. Circulation.

[4] Al-Khatib S.M., Stevenson W.G., Ackerman M.J., Bryant W.J., Callans D.J., Curtis A.B., Page R.L. (2018). 2017 AHA/ACC/HRS Guideline for Management of Patients with Ventricular Arrhythmias and the Prevention of Sudden Cardiac Death: A Report of the American College of Cardiology/American Heart Association Task Force on Clinical Practice Guidelines and the Heart Rhythm Society. Circulation.

[5] Wichter T., Paul M., Wollmann C., Acil T., Gerdes P., Ashraf O., Tjan T.D.T., Soeparwata R., Block M., Borggrefe M. (2004). Implantable Cardioverter/Defibrillator Therapy in Arrhythmogenic Right Ventricular Cardiomyopathy: Single-Center Experience of Long-Term Follow-Up and Complications in 60 Patients. Circulation.

[6] Kossaify A. (2020). Sensing and Detection Functions in Implantable Cardioverter Defibrillators: The Good, the Bad and the Ugly. Acta Cardiol. Sin..

[7] Hsu S.S., Mohib S., Schroeder A., Deger F.T. (2004). T wave oversensing in implantable cardioverter defibrillators. J. Interv. Card. Electrophysiol..

[8] Watanabe H., Chinushi M., Izumi D., Sato A., Okada S., Okamura K., Aizawa Y. (2006). Decrease in Amplitude of Intracardiac Ventricular Electrogram and Inappropriate Therapy in Patients with an Implantable Cardioverter Defibrillator. Int. Heart J..

[9] Mugnai G., Tomei R., Dugo C., Tomasi L., Morani G., Vassanelli C. (2014). Implantable cardioverter-defibrillators in patients with arrhythmogenic right ventricular cardiomyopathy: The course of electronic parameters, clinical features, and complications during long-term follow-up. J. Interv. Card. Electrophysiol..

[10] Christensen A.H., Platonov P.G., Svensson A., Jensen H.K., Rootwelt-Norberg C., Dahlberg P., Svendsen J.H. (2022). Complications of implantable cardioverter-defibrillator treatment in arrhythmogenic right ventricular cardiomyopathy. EP Eur..

[11] Lillo-Castellano J.M., Marina-Breysse M., Gómez-Gallanti A., Martinez-Ferrer J.B., Alzueta J., Pérez-Álvarez L., Filgueiras-Rama D. (2016). Safety threshold of R-wave amplitudes in patients with implantable cardioverter defibrillator. Heart.

[12] Aquaro G.D., De Luca A., Cappelletto C., Raimondi F., Bianco F., Botto N., Sinagra G. (2020). Prognostic value of magnetic resonance phenotype in patients with arrhythmogenic right ventricular cardiomyopathy. J. Am. Coll. Cardiol..

[13] Farrelly C., Shah S., Davarpanah A., Keeling A.N., Carr J.C. (2012). ECG-gated multiecho Dixon fat-water separation in cardiac MRI: Advantages over conventional fat-saturated imaging. Am. J. Roentgenol..

[14] Oda S., Morita K., Kidoh M., Nagayama Y., Nakaura T., Shirahama Y., Hirai T. (2021). Three-Dimensional Modified Dixon ECG-Gated Cardiac Magnetic Resonance Imaging in Arrhythmogenic Right Ventricular Cardiomyopathy/Dysplasia. Circ. Cardiovasc. Imaging.

[15] Jain A., Tandri H., Calkins H., Bluemke D.A. (2008). Role of cardiovascular magnetic resonance imaging in arrhythmogenic right ventricular dysplasia. J. Cardiovasc. Magn. Reson..

[16] Chen X., Li L., Cheng H., Song Y., Ji K., Chen L., Zhao S. (2019). Early left ventricular involvement detected by cardiovascular magnetic resonance feature tracking in arrhythmogenic right ventricular cardiomyopathy: The effects of left ventricular late gadolinium enhancement and right ventricular dysfunction. J. Am. Heart Assoc..

[17] Durbin J., Watson G.S. (1971). Testing for serial correlation in least squares regression. III. Biometrika.

[18] Bosman L.P., Nielsen Gerlach C.L., Cadrin-Tourigny J., Orgeron G., Tichnell C., Murray B., Te Riele A.S. (2022). Comparing clinical performance of current implantable cardioverter-defibrillator implantation recommendations in arrhythmogenic right ventricular cardiomyopathy. EP Eur..

[19] Link M.S., Wang P.J., Haugh C.J., Homoud M.K., Foote C.B., Costeas X.B., Estes N.A. (1997). Arrhythmogenic right ventricular dysplasia: Clinical results with implantable cardioverter defibrillators. J. Interv. Card. Electrophysiol..

[20] Breithardt G., Wichter T., Haverkamp W., Borggrefe M., Block M., Hammel D., Scheld H.H. (1994). Implantable cardioverter defibrillator therapy in patients with arrhythmogenic right ventricular cardiomyopathy, long QT syndrome, or no structural heart disease. Am. Heart J..

[21] Herman A.R., Gardner M., Steinberg C., Yeung-Lai-Wah J.A., Healey J.S., Leong-Sit P., Chakrabarti S. (2016). Long-term right ventricular implantable cardioverter-defibrillator lead performance in arrhythmogenic right ventricular cardiomyopathy. Heart Rhythm..

[22] Bonou M., Mavrogeni S., Kapelios C.J., Markousis-Mavrogenis G., Aggeli C., Cholongitas E., Barbetseas J. (2021). Cardiac Adiposity and Arrhythmias: The Role of Imaging. Diagnostics.

[23] Bucius P., Erley J., Tanacli R., Zieschang V., Giusca S., Korosoglou G., Kelle S. (2019). Comparison of feature tracking, fast-SENC, and myocardial tagging for global and segmental left ventricular strain. ESC Heart Fail..

[24] Bourfiss M., Prakken N.H.J., James C.A., Planken R.N., Boekholdt S.M., Ahmetagic D., Te Riele A.S.J.M. (2022). Prognostic value of strain by feature-tracking cardiac magnetic resonance in arrhythmogenic right ventricular cardiomyopathy. Eur. Heart J. Cardiovasc. Imaging.

[25] Kuschyk J., Müller-Leisse J., Duncker D., Tülümen E., Fastenrath F., Fastner C., Rudic B. (2021). Comparison of transvenous vs. subcutaneous defibrillator therapy in patients with cardiac arrhythmia syndromes and genetic cardiomyopathies. Int. J. Cardiol..

[26] Kempa M., Łaskawski G., Budrejko S., Królak T., Kozłowski D., Rogowski J., Raczak G. (2017). Epicardial screw-in sensing lead on the left ventricle to treat undersensing of ventricular arrhythmias in a patient with arrhythmogenic right ventricular cardiomyopathy. Cardiol. J..

[27] Taleski J. (2019). Left Ventricular Lead Placement for Pacing and Sensing in a Patient with Arrhythmogenic Right Ventricular Cardiomyopathy Undergoing ICD Implantation. Acta Clin. Croat..

[28] Lochy S., Francois B., Hollanders G., Provenier F. (2010). Left ventricular sensing and pacing for sensing difficulties in internal cardioverter defibrillator therapy for arrhythmogenic right ventricular cardiomyopathy. Europace.

[29] Augustine D., Lewandowski A.J., Lazdam M., Rai A., Francis J., Myerson S., Leeson P. (2013). Global and regional left ventricular myocardial deformation measures by magnetic resonance feature tracking in healthy volunteers: Comparison with tagging and relevance of gender. J. Cardiovasc. Magn. Reson..

