# Supplementary material for: Radial and Circumferential CMR-Based RV Strain Predicts Low R Wave Amplitude after ICD Implantation in Patients with Arrhythmogenic Cardiomyopathy
Source: J Clin Med. 2023 Jan 22;12(3):886. doi: 10.3390/jcm12030886 (PMC9917584; doi:10.3390/jcm12030886)
Supplement: Supplementary file 1 [file jcm-12-00886-s001.zip › jcm-2150737-supplementary.pdf]

Supplementary Materials:

Figure S1. An example of mDixon images for RV fat identification.

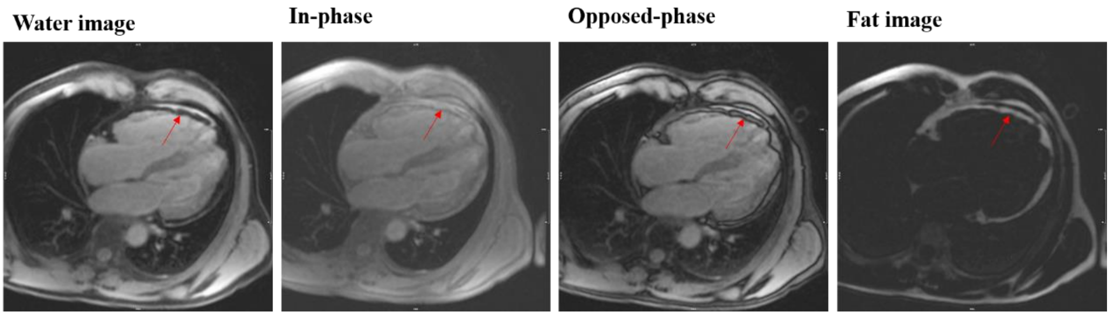

Water images show dilatation of the right ventricle (RV) and a scalloped appearance of the RV-free wall (red arrows). Fatty signal is evident the RV free wall on fat images and in-phase (both high signals), as well as opposed-phase, and water images (both low signals).

Figure S2. Relationship between R wave amplitude, ACM subtypes, lead position, ICD brands, and pacing parameters.

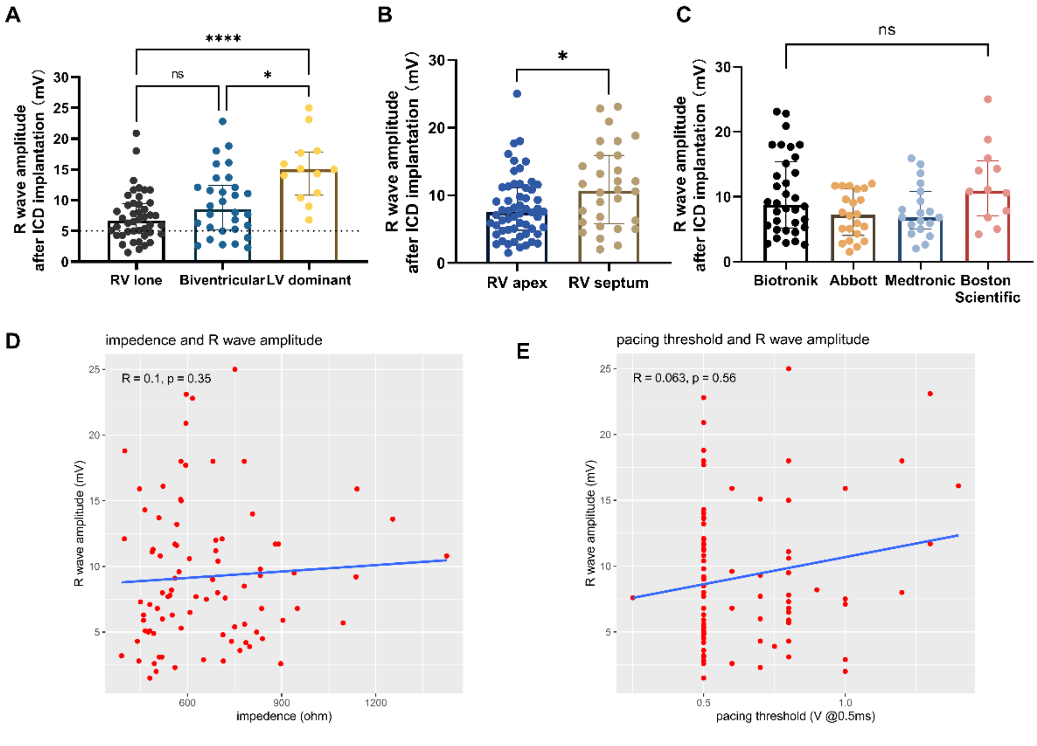

A. Comparison of R wave amplitude (RWA) between different ACM subgroups. B. Comparison of R wave amplitude (RWA) between groups with apical and septal lead placement. C. Comparison of RWA between different ICD brands. D: Correlation between RWA and pacing impedance, E: Correlation between RWA and pacing threshold. * Represents a p-value lower than 0.05. **** represents a p value lower than 0.0001, and ns represents non-significant.

Figure S3. Relationship between left ventricular parameters and R wave amplitude.

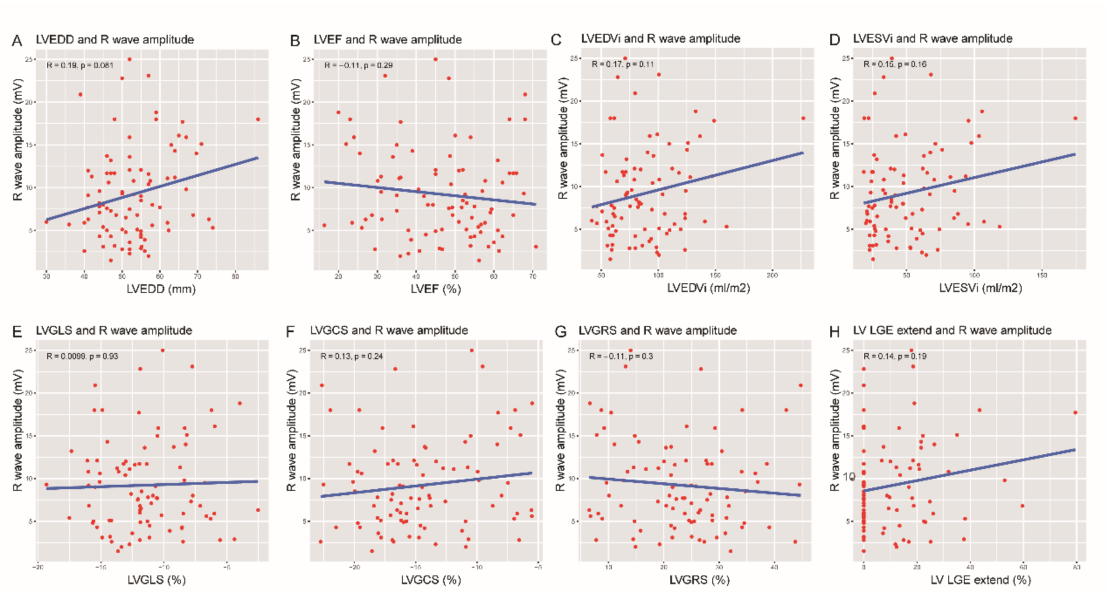

A–D: Correlation between left ventricular (LV) end-diastolic diameter, LV ejection fraction, LV end-diastolic volume index, LV end-systolic volume index, and R wave amplitude (RWA) of ICD lead.
E–H: Correlation between LV global longitudinal, circumferential, radial strain, LV-scar (LGE) extend, and RWA.

Figure S4. Changes of pacing parameters at 2–6-month follow-up and receiver operating curves of RV parameters and low RWA at 2–6 months.

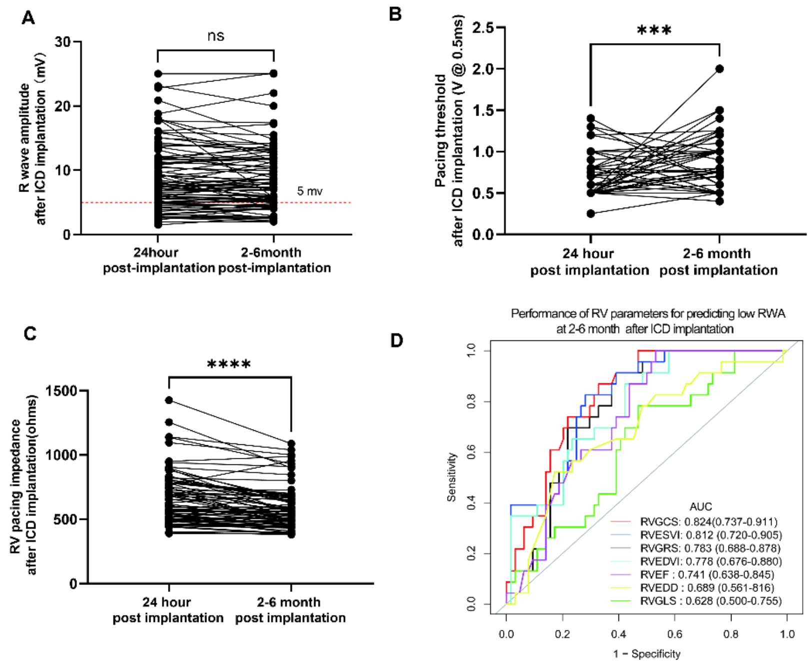

A–C. Comparison of R wave amplitude (RWA), pacing threshold and pacing impedance within 24 hours and at 2–6 month follow-up after ICD implantation. D. Receiver operating curves showing the predictive performance of RV parameters in identifying patients with low RWA at 2–6-month follow-up after ICD implantation in ACM patients. *** Represents a p-value lower than 0.001, **** represents a p value lower than 0.0001, and ns represents nonsignificant.

Table S1. Imaging parameters for predicting Low RWA in LV involvement subgroup.

	ROC area (AUC)	95%CI low	95%CI upper	cut-off	Sensitivity	Specificity
RVEDD, mm	0.854	0.675	1.000	41.8	0.833	0.703
RVEF, %	0.775	0.634	0.926	31.5	1.000	0.541
RVEDVi, ml/m^2	0.833	0.704	0.962	88.5	1.000	0.459
RVESVi, ml/m^2	0.842	0.717	0.968	78.4	1.000	0.676
RVGRS, %	0.860	0.719	1.000	8.75	0.833	0.703
RVGCS, %	0.892	0.760	1.000	-6.55	1.000	0.649
RVGLS, %	0.671	0.468	0.874	-11.43	0.833	0.568

Notes: RVEDD: right ventricular end diastolic diameter; RVEF: right ventricular ejection fraction; RVEDVi: right ventricular end diastolic volume index; RVESVi; right ventricular end-systolic volume index; RVGL (C/R) S; right ventricular global longitudinal, circumferential, radial strain; AUC: Area under receiver operative curve; CI: confidence interval. The cutoff was set according to the best cutoff used in the total population.

Table S2. Reproducibility for CMR-derived novel right ventricular strain parameters.

	Intra-observer			Inter-observer		
	ICC	95%CI	p-value	ICC	95%CI	p-value
RVGRS	0.886	0.699-0.960	<0.001	0.864	0.642-0.952	<0.001
RVGCS	0.889	0.731-0.965	<0.001	0.880	0.685-0.958	<0.001
RVGLS	0.923	0.785-0.973	<0.001	0.917	0.775-0.971	<0.001

Notes: RVGL (C/R) S: right ventricular global longitudinal, circumferential, radial strain; ICC: intraclass correlation coefficient.

Table S3. Correlation between right ventricular (RV) and left ventricular (LV) metrics and R wave amplitude (RWA) at 2–6-month follow-up.

Parameters	RV metrics Spearman's correlation coefficient	p-value	Parameters	LV metrics Spearman's correlation coefficient	p-value
RVGRS	0.63	<0.001	LVGRS	-0.20	0.065
RVGCS	-0.66	<0.001	LVGCS	0.12	0.275
RVGLS	-0.25	0.02	LVGLS	-0.01	0.895
RVESVI	-0.58	<0.001	LVESVI	0.15	0.175
RVEDVI	-0.54	<0.001	LVEDVI	0.15	0.181
RVEDD	-0.43	<0.001	LVEDD	0.19	0.071
RVEF	0.49	<0.001	LVEF	-0.12	0.289

Notes: RVEDD: right ventricular end diastolic diameter; RVEF: right ventricular ejection fraction; RVEDVi:right ventricular end diastolic volume index; RVESVi: right ventricular end-systolic volume index; RVGL (C/R) S: right ventricular global longitudinal, circumferential, radial strain; LVEDD: left ventricular end-diastolic diameter; LVEF: left ventricular ejection fraction; LVEDVi: left ventricular end-diastolic volume index; LVESVi: left ventricular end-systolic volume index; LVGL (C/R) S: left ventricular global longitudinal, circumferential, radial strain.
